# Evaluation of Subchronic Toxicity and Genotoxicity of Ethanolic Extract of *Aster glehni* Leaves and Stems

**DOI:** 10.1155/2021/1018101

**Published:** 2021-12-30

**Authors:** Mi Kyung Lim, Ju Yeon Kim, Jeongho Jeong, Eun Hye Han, Sang Ho Lee, Soyeon Lee, Sun-Don Kim, Jinu Lee

**Affiliations:** ^1^College of Pharmacy, Yonsei Institute of Pharmaceutical Sciences, Yonsei University, Incheon 21983, Republic of Korea; ^2^R&D Center, Koreaeundan Healthcare Co., Ltd., Ansan 15405, Republic of Korea; ^3^Safety Evaluation Center, Nonclinical Research Institute, ChemOn Inc., Yongin 17162, Republic of Korea

## Abstract

*Aster glehni*, a traditional plant on Ulleung Island in the Republic of Korea, has been recognized for its multiple medicinal properties. However, potential toxicity and safety analyses of *A. glehni* have not been previously investigated. Therefore, this study aimed to evaluate the safety profile of ethanolic extract of *A. glehni* leaves and stems (EAG) in terms of genotoxicity and subchronic oral animal toxicity under OECD guidelines and GLP conditions. Toxicological assessments were performed at doses of 1,250, 2,500, and 5,000 mg/kg/day in a 13-week oral repeated-dose toxicity study of EAG in male and female SD rats. In addition, an Ames test, an *in vitro* mammalian chromosomal aberration test, and a micronucleus test were performed. No toxicological changes in clinical signs, body weights, water and food consumption, urinalysis, hematology, clinical biochemistry, gross findings, and histopathological examinations were observed in subchronic oral animal toxicity. In addition, EAG gave negative results when evaluated using *in vitro* and *in vivo* genotoxicity tests. In conclusion, the no-observed-adverse-effect level (NOAEL) of EAG was considered to be 5,000 mg/kg/day, and no target organs were identified in both sexes of rats. EAG was also classified as nonmutagenic and nonclastogenic in genotoxicity testing. Collectively, these results show a lack of general toxicity and genotoxicity for EAG that supports clinical work for development as a herbal medicine.

## 1. Introduction

Medicinal plants have been used traditionally therapeutic agents, but recently, they have seen more and more as substitutes for chemical agents with side effects or drug resistance [1, 2]. Herbal medicine derived from medicinal plants often has anti-oxidant, anti-microbial, and anti-inflammatory properties so they may provide potential options for the treatment of diseases such as COVID-19 that yet has no approved drug [3–7]. Medicinal plants are utilized for the treatment of various diseases based on their unique biological properties such as anti-cancer, thrombolytic, and gastrointestinal function control, as well as for the improvement of neurological diseases by anti-nociceptive, anti-depressant, and anxiolytic activity [1, 2, 8–15]. However, some medicinal plants have reported toxicity like hepatotoxicity and renal toxicity at high doses and long-term use [5, 6, 16]. Therefore, it is essential to evaluate toxicity profiles for human safety.

*Aster glehni* Fr. Schm., widely distributed on Ulleung Island, Republic of Korea, is known to be a traditional edible herb. In a Korean traditional medical encyclopedia known as *Dongui Bogam*, it is described that *A. glehni* has anti-pyretic and analgesic effects and suppresses phlegm and coughing [17]. *A. glehni* has been used for the treatment of a variety of diseases including diabetes mellitus, hypercholesterolemia, and cardiovascular disease [18]. In addition, it has been reported that ethanolic extract of *A. glehni* shows anti-adipogenic, hypouricemic, and anti-inflammatory effects [18–20].

*A. glehni* contains caffeoylquinic acid (CQ) derivatives such as 3,5-di-O-caffeoylquinic acid (3,5-DCQA), 5-O-caffeoylquinic acid, 3-O-caffeoylquinic acid, and 3-O-*p*-coumaroylquinic acid and flavonoids such as astragalin and kaempferol [18, 21]. According to recent research, ethanolic extract of *A. glehni* (EAG) and 3,5-DCQA have ameliorating effects on memory impairment caused by scopolamine in male ICR mice [22, 23]. It has also been reported that 3,5-DCQA inhibits the activity of acetylcholinesterase (AChE) and amyloid-beta (A*β*) induced cytotoxicity in SH-SY5Y neuroblastoma cells [23–25]. These results suggest that 3,5-DCQA might play an important role in the ameliorative effects of EAG on memory dysfunction.

Although effects of EAG have been generally extensively perceived to be therapeutic, to date, adverse effects of EAG use in humans have not been reported. Thus, the present study of EAG was designed under Regulation on Approval and Notification of Herbal (crude) Medicinal Preparation, Etc. of the Korea Ministry of Food and Drug Safety (MFDS) [26] to provide safety information for a subsequent clinical trial. The toxicity studies of EAG were conducted as 2-week and 13-week repeated-dose oral toxicity tests in SD rats and genotoxicity tests following the Good Laboratory Practice regulations of the Organization for Economic Cooperation and Development [27] and MFDS [28].

## 2. Materials and Methods

### 2.1. Preparation of Ethanolic Extract of Aerial Parts of *A. glehni*

The aerial parts, leaves, and stems, of *A. glehni* were collected from Ulleung Island and dried naturally. The EAG was prepared by the method as previously described [23]. Briefly, the finely chopped sample was extracted in a 15-fold mass of 70% ethanol. The first extract was collected; then the second extract was obtained in a 10-fold mass of 70% ethanol. After mixing with diatomite, it was filtered and concentrated to 10–20 Brix. After adding an equal amount of dextrin to the concentrates, the mixtures were sterilized at 95°C for 30 min. The sterilized samples were spray-dried and filtered through a 60-mesh sieve to obtain a solid extract powder (Specimen Voucher No. AG-D022). For quality assurance, the final *A. glehni* extract was standardized by 3,5-dicaffeoylquinic acid (3,5-DCQA) based on high-performance liquid chromatography (HPLC) at 330 nm. The content of the marker compound (3,5-DCQA) in the EAG was 2.37 mg/g. The results of the amino acid composition analysis are shown in Table 1. The total protein content in EAG was 1,759.84 mg/100 g. Proline (35.1%), aspartic acid (21.6%), and glutamic acid (11.2%) were the main amino acids existing in EAG.

### 2.2. Experimental Animals and Maintenance

Specific pathogen-free (SPF) SD rats were obtained from Orient Bio Inc. (Seongnam, Korea). The animals were maintained in the facility with temperature (23 ± 3°C), relative humidity (55 ± 15%), and ventilation (10–20 air changes/hour) at Chemon Inc. in accordance with the Guide for the Care and Use of Laboratory Animals, 8th edition [29]. Food and water were provided, ad libitum, with a 12 hours light:12 hours dark cycle. All procedures and protocols were reviewed and approved by the Institutional Animal Care and Use Committee (IACUC) of Chemon Inc. performed in accordance with the guideline published by the Organization for Economic Cooperation and Development (OECD) as well as the Good Laboratory Practice (GLP) regulations for Nonclinical Laboratory Studies of the Ministry of Food Drug Safety (MFDS) in the Republic of Korea [27, 28, 30, 31].

### 2.3. Thirteen-Week Repeated Oral Toxicity Study

For the 13-week repeat-dose toxicity study, in accordance with OECD Guideline 408 [32], healthy 6-week old male and female SD rats weighing 186.56 ± 8.70 g and 144.41 ± 7.63 g, respectively, were randomly assigned to 4 groups (10/sex/group) under GLP regulations. Vehicle (distilled water for injection) or graded doses of EAG (1,250, 2,500, and 5,000 mg/kg according to body weight) were administered to rats by oral gavage once daily for 13 weeks at a dose of 10 mL/kg of body weight after completion of a 14-day repeated oral toxicity dose range finding (DRF) study where no adverse finding was seen dosing up to 5,000 mg/kg/day. The high dose was selected according to the results of an acute toxicity study in which no significant test article-related changes in mortalities and clinical signs at 5,000 mg/kg/day were observed (data not shown). The rats were observed daily for clinical signs including mortality, general appearance, and behavioral abnormality until terminal sacrifice. Body weights and food/water consumption were recorded weekly throughout the study. Ophthalmological examination was conducted in the last week of observation and anterior parts of the eye, optic media, and fundus were examined with a fundus camera (Vantage Plus Digital LED; Keeler Instruments Inc., Malvern, PA, USA). At study termination, all rats were euthanized by isoflurane (2% to 5%) inhalation for blood sample collection.

### 2.4. Urinalysis, Hematology, and Clinical Biochemistry

Urinalysis, hematological analyses, and serum biochemistry analyses were conducted as described previously [31].

### 2.5. Gross Findings, Organ Weights, and Histopathological Examinations

At necropsy, the animals were sacrificed to analyze both macroscopic and microscopic features of the internal organs. The organ weights were measured as described previously [31]. All tissues from each animal were preserved, and lesions were graded using a five-step scale in the order of increasing severity (minimal, mild, moderate, severe, and massive). Brain, jejunum, peripheral nerve, pituitary gland, ileum, femorotibial joint, lung, cecum, urinary bladder, heart, colon, testis, thymus, rectum, epididymis, spleen, eye with optic nerve, prostate gland, adrenal gland, thyroid gland with parathyroid gland, seminal vesicle with coagulating gland, kidney, Harderian gland, ovary, liver, salivary gland, uterus with cervix, tongue, aorta, vagina, trachea, sternum with bone marrow, skin, esophagus, mandibular lymph node, mammary gland, stomach, mesenteric lymph node, skeletal muscle, pancreas, thoracic spinal cord, gross lesion, and duodenum were processed for histopathological examination using Pristima® (Xybion, Lawrenceville, NJ, USA). Diagnostic terms in the Lexicon of Pristima® were used primarily. Standardized System of Nomenclature and Diagnostic Criteria-Guides for Toxicologic Pathology [33] and Covance Glossary [34] were also utilized.

### 2.6. Bacterial Reverse Mutation Assay

Four histidine auxotroph strains of *Salmonella typhimurium* (TA100, TA1535, TA98, and TA1537) [35] and a tryptophan auxotroph strain of *Escherichia coli* WP2 *uvr*A [36] were used to confirm mutagenicity of EAG according to OECD Guideline 471 [37] under GLP conditions. The mutagenic activity of EAG was assessed both in the presence and absence of an external metabolic activation system from rat livers (S9 fraction) using the direct plate incorporation method. For the plating assay, 0.5 mL of S9 mix (or sodium phosphate buffer, pH 7.4 for nonactivation plates), 0.1 mL of bacterial culture (containing approximately 10^8^ viable cells), and 0.1 mL of test article were mixed with 2.0 mL of overlay agar. The contents of each tube were mixed and poured over the surface of a minimal agar plate. The overlay agar was allowed to solidify before incubation. After the top layers solidified, plates were inverted and incubated at 37 ± 2°C for 50 ± 2 h and revertant colonies were counted by the unaided eye. EAG was applied at dose levels of 50, 150, 500, 1,500, 3,000, and 5,000 *µ*g/plate. The positive control substances were 2-aminoanthracene (2-AA), benzo[a]pyrene (B[a]P), sodium azide (SA), 2-nitrofluorene (2-NF), acridine mutagen ICR 191 (ICR-191), and 4-nitroquinoline-1-oxide (4NQO). At least three independent experiments were performed using triplicate plates for each concentration. Results are expressed as revertant colonies and mutagenic indexes (MI).

### 2.7. In Vitro Chromosomal Aberration Test

A chromosomal aberration test was performed to evaluate the mutagenic potential to induce structural and/or numerical chromosomal aberrations in a CHL/IU cell line derived from the lung of a female Chinese hamster lung fibroblasts under OECD Guideline 473 [38]. The treatment methods were classified under three types according to the presence and absence of the metabolic active system. Treatment 1 was performed for 6 h using a metabolic activation system (S9 mix), and 18 h of recovery time was allowed to observe the chromosomal aberrations. Treatments 2 and 3 were performed for 6 h and 24 h, respectively, without the use of S9 mix and followed by an 18 h and 0 h recovery, respectively. In Treatment 1, EAG was used at concentrations of 0 (negative control), 350, 700, 1,300, and 1,400 *µ*g/mL. Treatments 2 and 3 were applied at 0, 300, 600, 1,100, and 1,200 *µ*g/mL and 0, 225, 450, 800, and 900 *µ*g/mL, respectively. Approximately 22 hours after treatment, 50 *μ*L of colchicine solution was added to each culture (final concentration of 1 *μ*M) and incubated for 2 hours for mitotic arrest. The mitotic cells were detached by gentle shaking. The media containing mitotic cells were centrifuged, and the cell pellets were resuspended in 75 mM potassium chloride solution for hypotonic treatment. Then cells were fixed with fixative (methanol: glacial acetic acid = 3:1 v/v), and slides were prepared by the air-drying method. Slides were stained with 5% Giemsa solution. Two slides were prepared for each culture. The results were expressed as frequency (%) of metaphases with structural or numerical aberrations per 300 metaphases. The relative increase in cell count (RICC %) was used as an indicator of concurrent cytotoxicity to determine the high concentration. With the cell counts, RICC (%) was calculated as follows:

(1) $$\begin{array}{c} \text{RICC}\left(\%\right)=\frac{\left(\text{Cell count of treated flask}-\text{Initial cell count}\right)}{\left(\text{Cell count of control flask}-\text{Initial cell count}\right)}\times 100. \end{array}$$

### 2.8. In Vivo Micronucleus Test in Mammalian Bone Marrow

Eight-week-old male ICR mice, 35.3 ± 1.3 g, were administered orally once a day for two consecutive days at doses of 500, 1,000, and 2,000 mg/kg/day (*n* = 6 in each group) according to OECD Guideline 474 [39]. Sterile distilled water for injection (10 mL/kg) was used as a negative control. Cyclophosphamide monohydrate (CPA) 70 mg/kg was administered once intraperitoneally on the day of the second administration as a positive control. All mice were daily observed for clinical signs. All animals were sacrificed about 24 h after the final administration, and bone marrow preparations were made for the evaluation of micronuclei and cytotoxicity. The bone marrow cells were fixed with methanol according to the method described in Schmid [40] and stained with acridine orange prepared based on the method of Hayashi [41]. The cells were observed and counted using a fluorescence microscope, and the identification of micronuclei was confirmed by the method of Hayashi [41]. Micronucleated polychromatic erythrocytes (MNPCE) were counted among 4,000 polychromatic erythrocytes (PCE) per animal. The ratio of PCE to total erythrocytes (red blood cell), an indicator of cytotoxicity [42], was determined by counting 500 erythrocytes per animal.

### 2.9. Statistical Analysis

SPSS Statistics 22 for Medical Science was used for all statistical analyzes, and the level of significance was *P* < 0.05. Body weights, food and water consumption, urine volume, hematological and clinical biochemistry parameters, and organ weights were assumed to be normally distributed and analyzed by parametric one-way analysis of variance (ANOVA). The assumption of homogeneity was tested using Levene's test. The urinalysis data were rank-transformed and analyzed by the nonparametric Kruskal–Wallis H test. Fisher's exact test was used to compare the frequency of aberrant metaphases between the negative control and test article-treated groups in the chromosomal aberration test. In the micronucleus test, the frequency of micronucleus was analyzed by the nonparametric Kruskal–Wallis H test. The negative and positive control groups were compared by the Mann–Whitney U test. The dose-responsiveness was tested by the linear-by-linear association of the chi-square test. The PCE:RBC ratio was assumed to be normally distributed and analyzed by one-way ANOVA, and the assumption of homogeneity of variance was tested using Levene's test. The Student's *t*-test was used to test for a difference between means of the negative and positive control.

## 3. Results

### 3.1. Thirteen-Week Repeated Oral Toxicity Study

**Figure 1:**
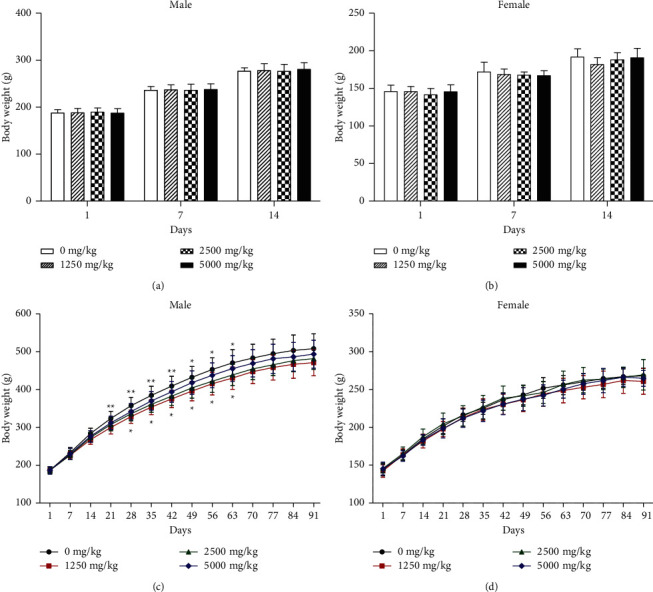
Effect of ethanolic extract of *A. glehni* on body weights in SD rats. (a) Mean body weights of male rats and (b) mean body weight of female rats treated with EAG for 2 weeks. (c) Mean body weights of male rats and (d) mean body weight of female rats treated with EAG for 13 weeks. Values are expressed as mean ± SD (*n* = 9–10 per group). Significant difference at ^*∗*^*P* < 0.05 and ^∗∗^*P* < 0.01 levels compared with the negative control.

There is still insufficient toxicological information on the oral toxicity of EAG after long-term exposure. Therefore, a repeated-dose toxicity DRF study of EAG at doses of 1,250, 2,500, and 5,000 mg/kg/day administered by oral gavage for 14 days was performed to assess initial toxicity. As a result, no EAG-related changes in mortalities, clinical signs, body weights, food and water consumption, ophthalmological examination, urinalysis, hematological and clinical biochemistry tests, organ weight, and gross findings were observed during the 2-week treatment period (body weights as shown in Figure 1 and other data not shown).

In the 13-week repeated-dose toxicity study, although one male rat treated with 1,250 mg/kg/day of EAG died on day 65, there were no clinical signs or any lesions in histopathological examination. The compound-colored stool was observed at 5,000 mg/kg/day in both sexes from day 10 to necropsy day, and salivation was sporadically observed in males at 5,000 mg/kg/day. Significant decreases in mean body weight were observed in males at 1,250 and 2,500 mg/kg/day (*P* < 0.05 and *P* < 0.01; Figure 1), but these changes did not occur in a dose-dependent manner, and the values were within the normal physiological ranges [43, 44]. No significant changes were found in female body weight between the treatment and control groups. There were no EAG-related effects in food intake, water intake, organ weights, and ophthalmological test in both sexes (data not shown).

A few instances of mean values of urinalysis parameters differing with statistical significance from the negative control were observed (*P* < 0.05 and *P* < 0.01; Table 2). Ketone body in males at 5,000 mg/kg/day and specific gravity at all doses in females was significantly higher than that of the negative control. In addition, pH in females at all EAG groups was significantly higher and 24 hours total volume of urine in females at 1,250 and 5,000 mg/kg/day were significantly lower than those of negative control. However, these changes were within the normal physiological ranges [43, 44]. Therefore, these observations were not considered to be toxicologically significant.

Hematology evaluation showed lymphocyte count at 5,000 mg/kg/day in males was significantly higher, and prothrombin time at all doses in females was significantly lower compared with the negative control (*P* < 0.05 and *P* < 0.01; Table 3). However, these results were also within the normal physiological ranges [43, 44]. The results of the clinical biochemistry test were presented in Table 4. EAG-related changes in clinical biochemistry parameters were not found in both sexes.

In histopathological examinations, notable change was observed in the nonglandular stomach (Table 5). Squamous hyperplasia of the limiting ridge in the stomach was found in the EAG-treated groups in both sexes. The changes were observed in seven males at 2,500 mg/kg/day and in all males and eight females at 5,000 mg/kg/day (*P* < 0.01 and *P* < 0.001). However, there were no toxicologically significant changes in histopathological examinations. Other lesions that have been well known to occur spontaneously in the same age of SD rats were observed [45, 46].

### 3.2. Genotoxicity Test

#### 3.2.1. Bacterial Reverse Mutation Test (Ames Test)

No precipitation or other abnormality was observed on the bottom agar at the time of plate scoring. There was a dose-related increase in a number of colonies in TA98 that is one of the histidine-requiring strains at 3,000 and 5,000 *µ*g/plate, and the number of revertants was 2.3 and 2.5 times higher than that of the negative control in the presence of S9 mix, respectively (Table 6). However, EAG is composed of various amino acids, including histidine (Table 1). In other test strains, no substantial increases in numbers of revertants per plate were observed in any dose level of EAG. Moreover, there were no signs of cytotoxicity at any dose level in all test strains. The results suggest that EAG is not mutagenic in the test strains. The mean revertants in the positive control for each strain showed a clear increase over the mean revertants in the negative control for that strain.

#### 3.2.2. Chromosome Aberration Test Using CHL Cells

In this experiment, no turbidity or precipitation was observed at all dose levels of EAG. As shown in Table 7, there was no statistically significant increase at any dose level of EAG compared to the negative control, and there was no dose-response relationship or increase in the frequency of aberrant metaphases in all treatment series. In the positive control, there was a statistically significant increase in the mean frequency of aberrant metaphases with structural aberrations in all treatment series (*P* < 0.01).

#### 3.2.3. Micronucleus Test Using Mouse Bone Marrow Cells

One mouse at 500 mg/kg/day died after the first administration, but it was not considered to be EAG-related, and there were no noticeable macroscopic signs in all other survivors that could be attributed to EAG. There was no statistically significant increase or a dose-related increase in the frequencies of MNPCE at any dose level of EAG compared to the negative control (Table 8). The PCE:RBC ratio showed no difference at any dose level of EAG. In contrast, the micronucleus and PCE:RBC ratio were significantly changed by the positive control (*P* < 0.01) when compared to the negative control.

## 4. Discussion

Regarding the utilization of *A. glehni*, several studies have concentrated on its pharmacological and therapeutic effects on diverse diseases [17–21]. Recently, it has been reported that ethanolic extract of *A. glehni* has ameliorating effects on scopolamine-induced memory dysfunction including long-term or working memory and improves memory function [22]. According to the study, its effects are due to the inhibition of acetylcholinesterase activity and the activation of ERK-CREB-BDNF and PI3K-Akt-GSK-3*β* pathways [22, 23]. However, further application of *A. glehni* in herbal medicine or functional food has been limited as there is inadequate knowledge of its safety. The present study evaluated the potential toxicity of EAG after 2- and 13-week repeated oral administration in SD rats. Moreover, genotoxicity studies including bacterial reverse mutation assay, chromosomal aberration test, and micronucleus test in mammalian bone marrow were performed to investigate genotoxicity of EAG.

In the acute toxicity study, EAG was found to be nontoxic in rats, and the approximate lethal dose was higher than 5,000 mg/kg (data not shown). In the 2- and 13-week repeated oral toxicity studies, no changes by the test article were found in body weights, food and water consumption, organ weights, ophthalmological test, hematological test, and clinical biochemistry test. Although one male rat at 1,250 mg/kg/day died, there were no noticeable clinical signs or findings to ascertain the cause of death. Necropsy findings of retention of dark brown substance in the lung and the thoracic cavity were determined to be related to an administration error. In addition, the compound-colored stool was observed and considered to be related to EAG-treatment. However, this clinical sign was regarded as a change attributable to the excretion of EAG. Therefore, these findings were not considered to be adverse effects [47].

Urinalysis, involving an evaluation of renal functions, is often affected by test article toxicity and is evaluated by testing urinalysis parameters as an indirect indicator of kidney damage [48]. In urinalysis, significant changes were observed for the ketone body at 5,000 mg/kg/day in males and specific gravity at all doses in females under present experimental conditions. However, these results were not considered as toxic effects by EAG since the degree of changes were small and there were no related histopathological findings in the kidney. At necropsy, adhesion of irregular surface on the middle lobe of liver with the diaphragm was observed in female at 1,250 mg/kg/day and in male negative control. The irregular surface of the stomach and weak brown discoloration of the kidney were observed only once in males at 5,000 mg/kg/day. Retention of clear fluid was observed in all-female groups, and one female at 1,250 mg/kg/day exhibited dark red fluid. Other abnormalities, including partly black half spots on a glandular region of the stomach and nodule of ovary, were observed only once in females at 1,250 mg/kg/day and 2,500 mg/kg/day, respectively. All gross findings were microscopically confirmed as corresponding findings. However, these changes were not considered to be related to EAG because the incidence was low, there was no dose-response relationship, and they have been reported to be spontaneous and incidental [45, 46].

From the histopathological examination, EAG-related change was observed in the nonglandular region (limiting ridge) in the stomach. Squamous cell hyperplasia of limiting ridge in stomach increased in the EAG-treated groups in both sexes, and there was a dose-response relationship. However, the change was considered a nonreversible effect because the degree of the lesion was graded near mild and cellular atypia was not observed. In addition, it has been shown that if cellular atypia is not observed, squamous cell hyperplasia caused by ethyl acrylate can be recovered completely [45]. In addition, the forestomach is present only in rodents, and no EAG-related changes were observed in the other digestive organs including the glandular stomach. Therefore, toxicological significance was considered minimal. Consequently, the no-observed-adverse-effect level (NOAEL) was 5,000 mg/kg/day in both sexes, and no target organ was observed.

The genotoxic evaluation of EAG was tested by the Ames test, *in vitro* chromosomal aberration test in CHL cells, and *in vivo* mammalian micronucleus test. In the bacterial reverse mutation test, four histidine auxotroph strains of *S. typhimurium* and a tryptophan auxotroph strain of *E. coli* were tested both in the presence and absence of an exogenous metabolic activation system. The number of revertants did not increase at any dose level of EAG under present experimental conditions except for the TA98 strain. In the TA98 strain in the presence of S9 mix, there was a dose-related increase in the number of revertants, and the increase was reproducible. However, it was confirmed that EAG contains histidine so the results of the Ames test were deemed inconclusive. As an additional test for histidine content, two test articles were evaluated: leaf extract (histidine content 39.52 mg/100 g) and leaf and stem extract (histidine content 16.69 mg/100 g) of *A. glehni* at 5,000 *µ*g/plate. The number of revertants of leaf extract of *A. glehni* increased about 1.5-fold compared to leaf and stem extract of *A. glehni* so the finding in TA98 was related to the amount of the histidine content. It has been reported that the increases of revertants in the Ames test could be caused by a test article including histidine [49]. Histidine compounds can generate additional background growth of *S. typhimurium* on minimal medium plates, thereby resulting in spontaneous his^+^ revertants [50, 51]. Besides, it was noted that plants and their metabolites have possibly caused false positives by containing histidine [52–54]. Therefore, the result of revertants in TA98 strain was assessed to be a false positive due to histidine content in EAG.

For the chromosome aberration test, CHL cells were used to investigate the potential to induce chromosomal aberrations both in the presence and absence of an exogenous metabolic activation system. The result was regarded as clear negative if there was no statistically significant increase in the frequencies of aberrant metaphases at any dose level compared to the negative control, and there was no dose-response relationship or increase in the frequency of aberrant metaphases in all treatment series. The results met the criteria so EAG was clearly negative.

The results of the micronucleus test using mouse bone marrow cells showed that EAG did not induce any statistically significant increase or dose-related increase in the frequencies of MNPCE per 4,000 PCE at any dose level. In addition, there was no significant difference in the PCE:RBC ratio. The results indicate that EAG did not induce micronuclei in ICR mice mammalian bone marrow cells under present experimental conditions. Taken together, these results revealed that EAG was nongenotoxic in both *in vitro* and *in vivo* models.

## 5. Conclusion

This study assessed the safety of EAG using different model approaches, including subchronic oral toxicity studies and a battery of genotoxicity studies. When rats were given 2- or 13-week repeated-dose oral administration of EAG, at up to 5,000 mg/kg/day, the NOAEL was considered to be 5,000 mg/kg/day, and no target organs were identified in both sexes under present experimental conditions of this study. Moreover, EAG was also classified as nonmutagenic and nonclastogenic in genotoxicity testing. Collectively, these results show a lack of general toxicity and genotoxicity for EAG that supports clinical work for development as a herbal medicine.

## Figures and Tables

**Table 1:** Amino acid composition of EAG.

Amino acid^a^	Tyr	Gly	Ser	Ala	Glu	Lys	Leu	Met	Val	Arg	Asp	Ile	Thr	Phe	Pro	His	Cys	Trp
AG	ND	225.0	183.4	161.9	355.0	47.1	193.7	19.3	141.1	53.6	292.6	117.2	154.6	146.2	203.7	39.5	96.9	13.0
EAG	ND	53.9	78.3	67.9	197.6	7.1	84.6	ND	41.3	26.7	380.1	46.4	60.4	43.0	616.9	16.7	28.2	10.9

^a^Unit: mg/100 g, Tyr, tyrosine; Gly, glycine; Ser, serine; Ala, alanine; Glu, glutamic acid; Lys, lysine; Leu, leucine; Met, methionine; Val, valine; Arg, arginine; Asp, aspartic acid; Ile, isoleucine; Thr, threonine; Phe, phenylalanine; Pro, proline; His, histidine; Cys, cysteine; Trp, tryptophan; ND, not detected; AG, ethanolic leaf extract of *Aster glehni*; and EAG, ethanolic leaf and stem extract of *Aster glehni*.

**Table 2:** Urinalysis of male and female SD rats in the 13-week repeated oral toxicity study of EAG.

Tests	Result	EAG (mg/kg/day)							
		Male				Female			
		0	1,250	2,500	5,000	0	1,250	2,500	5,000
No. of animals examined		5	5	5	5	5	5	5	5
GLU	Negative	5	5	5	5	5	5	5	5
BIL	Negative	5	5	5	4	5	5	5	5
	Small	0	0	0	1	0	0	0	0

KET	Negative	3	1	4	1	5	5	4	4
	Trace	2	4	1	0	0	0	1	1
	15	0	0	0	3	0	0	0	0
	40	0	0	0	1^*∗*^	0	0	0	0

SG	≤1.005	1	0	0	0	5	1	1	0
	1.010	4	2	4	2	0	3	2	3
	1.015	0	2	1	2	0	1	1	1
	1.020	0	1	0	1	0	0^*∗*^	1^*∗*^	1^∗∗^

pH	6.5	0	0	0	0	1	0	0	0
	7.0	0	0	0	0	1	0	0	0
	7.5	1	0	0	0	3	1	0	0
	8.0	2	0	0	1	0	0	1	0
	8.5	2	5	5	4	0	4^*∗*^	4^∗∗^	5^∗∗^
Volume (mL)		13.0 ± 4.6	11.6 ± 1.9	15.2 ± 1.1	11.4 ± 4.4	17.6 ± 5.5	10.8 ± 2.8^*∗*^	12.4 ± 4.6	8.8 ± 4.0^∗∗^

^*∗*^/^∗∗^Significant difference at *P* < 0.05/*P* < 0.01 levels compared with the negative control by the Mann–Whitney *U* test. GLU, glucose (mg/dL); BIL, bilirubin (mg/dL); KET, ketone body (mg/dL); and SG, specific gravity.

**Table 3:** Hematological parameters of male and female SD rats in the 13-week repeated oral toxicity study of EAG.

Tests	EAG (mg/kg/day)			
	0	1,250	2,500	5,000
Male				
RBC (10^6^/*μ*L)	8.99 ± 0.55	9.01 ± 0.22^a^	8.81 ± 0.37	8.87 ± 0.42
HGB (g/dL)	15.3 ± 0.5	15.5 ± 0.4^a^	15.0 ± 0.5	15.0 ± 0.4
HCT (%)	47.3 ± 1.8	47.9 ± 1.4^a^	46.7 ± 1.3	46.6 ± 1.6
MCV (fL)	52.7 ± 1.9	53.2 ± 1.0^a^	53.1 ± 2.6	52.5 ± 1.1
MCH (pg)	17.1 ± 0.8	17.2 ± 0.3^a^	17.0 ± 1.0	16.9 ± 0.5
MCHC (g/dL)	32.4 ± 0.5	32.3 ± 0.4^a^	32.1 ± 0.8	32.3 ± 0.5
PLT (10^3^/*μ*L)	919.2 ± 61.3	905.9 ± 93.0^a^	890.4 ± 71.7	933.3 ± 74.8
WBC (10^3^/*μ*L)	6.30 ± 1.37	7.22 ± 2.18^a^	7.54 ± 1.16	7.91 ± 0.97
NEU (10^3^/*μ*L)	1.3 ± 0.3	1.5 ± 0.6^a^	1.6 ± 0.7	1.1 ± 0.2
LYM (10^3^/*μ*L)	4.6 ± 1.2	5.2 ± 1.6^a^	5.4 ± 1.0	6.3 ± 1.0^∗^
MONO (10^3^/*μ*L)	0.28 ± 0.12	0.31 ± 0.10^a^	0.32 ± 0.11	0.30 ± 0.06
EOS (10^3^/*μ*L)	0.11 ± 0.04	0.11 ± 0.03^a^	0.13 ± 0.02	0.10 ± 0.03
BASO (10^3^/*μ*L)	0.01 ± 0.01	0.01 ± 0.01^a^	0.01 ± 0.00	0.01 ± 0.00
PT (sec)	8.0 ± 0.2	8.1 ± 0.2^a^	8.0 ± 0.2	7.8 ± 0.2
Female				
RBC (10^6^/*μ*L)	7.98 ± 0.35	7.72 ± 0.30	7.86 ± 0.22	7.94 ± 0.28
HGB (g/dL)	14.3 ± 0.3	14.0 ± 0.4	14.1 ± 0.3	14.3 ± 0.4
HCT (%)	43.5 ± 1.3	42.8 ± 1.2	43.2 ± 1.9	43.7 ± 1.2
MCV (fL)	54.6 ± 1.8	55.5 ± 2.1	54.9 ± 0.8	55.0 ± 0.8
MCH (pg)	17.9 ± 0.6	18.1 ± 0.7	18.0 ± 0.4	17.9 ± 0.3
MCHC (g/dL)	32.8 ± 0.2	32.7 ± 0.4	32.7 ± 0.4	32.6 ± 0.4
PLT (10^3^/*μ*L)	969.9 ± 60.9	1023.9 ± 89.3	977.4 ± 87.8	950.3 ± 66.4
WBC (10^3^/*μ*L)	3.67 ± 0.95	3.75 ± 1.03	3.84 ± 1.22	4.01 ± 1.18
NEU (10^3^/*μ*L)	0.5 ± 0.1	0.5 ± 0.1	0.5 ± 0.2	0.5 ± 0.2
LYM (10^3^/*μ*L)	3.0 ± 0.9	3.0 ± 0.9	3.1 ± 1.0	3.3 ± 0.9
MONO (10^3^/*μ*L)	0.09 ± 0.04	0.11 ± 0.03	0.11 ± 0.04	0.13 ± 0.05
EOS (10^3^/*μ*L)	0.08 ± 0.03	0.08 ± 0.02	0.08 ± 0.03	0.07 ± 0.03
BASO (10^3^/*μ*L)	0.01 ± 0.01	0.00 ± 0.01	0.00 ± 0.00	0.00 ± 0.01
PT (sec)	7.7 ± 0.2	7.4 ± 0.2^##^	7.3 ± 0.2^##^	7.4 ± 0.2^##^

Data are expressed as mean ± standard deviation.^*∗*^Significant difference at *P* < 0.05 levels compared with the negative control by Scheffe multiple range test. ^##^Significant difference at *P* < 0.01 levels compared with the negative control by Duncan multiple range test. ^a^Number of animals in the group was 9; otherwise mean of 10 animals/sex/group. RBC, red blood cell; HGB, hemoglobin concentration; HCT, hematocrit; MCV, mean corpuscular volume; MCH, mean cell hemoglobin; MCHC, mean cell hemoglobin concentration; PLT, platelet count; WBC, white blood cell; NEU, neutrophil; LYM, lymphocyte; MONO, monocyte; EOS, eosinophil; BASO, basophil; and PT, prothrombin time.

**Table 4:** Clinical biochemistry parameters of male and female SD rats in the 13-week repeated oral toxicity study of EAG.

Tests	EAG (mg/kg/day)			
	0	1,250	2,500	5,000
Male				
AST (U/L)	83.7 ± 16.7	77.2 ± 14.9^a^	82.6 ± 15.9	70.6 ± 6.6
ALT (U/L)	33.3 ± 5.8	32.6 ± 6.3^a^	33.1 ± 4.1	31.8 ± 3.1
ALP (U/L)	88.1 ± 15.4	82.1 ± 16.0^a^	89.8 ± 17.8	93.5 ± 18.0
CPK (U/L)	160.9 ± 80.9	173.8 ± 124.2^a^	157.1 ± 94.7	118.0 ± 50.8
TBIL (mg/dL)	0.149 ± 0.030	0.145 ± 0.032^a^	0.145 ± 0.020	0.145 ± 0.020
GLU (mg/dL)	155.0 ± 19.3	149.7 ± 14.8^a^	151.1 ± 22.1	145.0 ± 17.0
TCHO (mg/dL)	89.0 ± 21.2	101.8 ± 21.3^a^	101.4 ± 24.0	104.8 ± 24.2
TG (mg/dL)	56.3 ± 25.8	63.2 ± 26.2^a^	60.6 ± 19.9	65.6 ± 28.2
TP (g/dL)	6.27 ± 0.16	6.37 ± 0.19^a^	6.29 ± 0.29	6.30 ± 0.26
ALB (g/dL)	2.90 ± 0.07	2.95 ± 0.11^a^	2.95 ± 0.11	2.93 ± 0.09
BUN (mg/dL)	13.9 ± 1.6	14.7 ± 1.1^a^	14.4 ± 2.3	13.6 ± 1.9
CRE (mg/dL)	0.40 ± 0.03	0.39 ± 0.02^a^	0.39 ± 0.02	0.40 ± 0.03

Female				
AST (U/L)	70.1 ± 11.2	76.8 ± 13.8	76.4 ± 14.2	73.0 ± 18.0
ALT (U/L)	22.1 ± 3.5	24.1 ± 6.3	25.0 ± 4.6	25.5 ± 3.6
ALP (U/L)	43.5 ± 15.6	54.2 ± 14.1	46.8 ± 13.4	45.8 ± 11.1
CPK (U/L)	146.6 ± 126.3	126.4 ± 84.9	149.6 ± 94.6	128.1 ± 54.7
TBIL (mg/dL)	0.169 ± 0.024	0.190 ± 0.038	0.176 ± 0.020	0.174 ± 0.018
GLU (mg/dL)	121.4 ± 14.5	129.3 ± 16.1	122.7 ± 14.6	122.0 ± 10.7
TCHO (mg/dL)	86.2 ± 20.0	92.5 ± 8.5	100.5 ± 17.6	85.3 ± 10.9
TG (mg/dL)	35.6 ± 6.3	35.0 ± 4.7	36.3 ± 8.4	32.9 ± 8.4
TP (g/dL)	5.89 ± 0.26	6.11 ± 0.21	6.02 ± 0.20	5.97 ± 0.17
ALB (g/dL)	2.99 ± 0.13	3.11 ± 0.15	3.05 ± 0.11	3.12 ± 0.11
BUN (mg/dL)	15.8 ± 2.3	15.0 ± 1.3	15.0 ± 2.4	14.3 ± 1.2
CRE (mg/dL)	0.48 ± 0.04	0.47 ± 0.03	0.49 ± 0.06	0.46 ± 0.02

Data are expressed as mean ± standard deviation. ^a^Number of animals in group was 9; otherwise mean of 10 animals/sex/group. AST, aspartate aminotransferase; ALT, alanine aminotransferase; ALP, alkaline phosphatase; CPK, creatine phosphokinase; TBIL, total bilirubin; GLU, glucose; TCHO, total cholesterol; TG, triglyceride; TP, total protein; ALB, albumin; BUN, blood urea nitrogen; and CRE, creatinine.

**Table 5:** Histopathologic findings of male and female SD rats in the 13-week repeated oral toxicity study of EAG.

Organs	Findings	EAG (mg/kg/day)							
		Male				Female			
		0	1,250	2,500	5,000	0	1,250	2,500	5,000
Nonglandular stomach	Hyperplasia, squamous cells, limiting ridge	0	3	7^∗∗^	10^∗∗∗^	0	1	3	8^∗∗∗^
No. of animals examined		10	10	10	10	10	10	10	10

^∗∗^/^∗∗∗^Significant difference at *P* < 0.01/*P* < 0.001 levels compared with the negative control by Fisher two-tailed test.

**Table 6:** Results of bacterial reverse mutation assay.

Test article	Dose (*μ*g/plate)	Colonies/plate [factor]^a^									
		TA98		TA100		TA1535		TA1537		WP2 *uvr*A	
		−S9 mix	+S9 mix	−S9 mix	+S9 mix	−S9 mix	+S9 mix	−S9 mix	+S9 mix	−S9 mix	+S9 mix
EAG	0	26 ± 4	25 ± 3	114 ± 8	107 ± 9	14 ± 2	15 ± 2	15 ± 2	15 ± 2	22 ± 3	29 ± 4
		—	—	—	—	—	—	—	—	—	—
	50	25 ± 6	28 ± 3	111 ± 6	102 ± 9	12 ± 4	16 ± 5	13 ± 2	13 ± 3	22 ± 4	25 ± 4
		[1.0]	[1.1]	[1.0]	[0.9]	[0.9]	[1.1]	[0.9]	[0.9]	[1.0]	[0.9]
	150	27 ± 4	30 ± 5	107 ± 15	120 ± 16	10 ± 1	15 ± 1	15 ± 1	12 ± 3	18 ± 3	26 ± 7
		[1.1]	[1.2]	[0.9]	[1.1]	[0.8]	[1.0]	[1.0]	[0.8]	[0.8]	[0.9]
	500	34 ± 5	34 ± 4	103 ± 7	105 ± 5	13 ± 1	17 ± 1	13 ± 1	14 ± 1	20 ± 2	26 ± 6
		[1.3]	[1.4]	[0.9]	[1.0]	[1.0]	[1.1]	[0.9]	[1.0]	[0.9]	[0.9]
	1,500	26 ± 5	40 ± 3	109 ± 6	120 ± 10	11 ± 1	15 ± 2	12 ± 2	13 ± 2	23 ± 4	26 ± 4
		[1.0]	[1.6]	[1.0]	[1.1]	[0.8]	[1.0]	[0.8]	[0.9]	[1.0]	[0.9]
	3,000	34 ± 5	56 ± 5	121 ± 2	137 ± 1	13 ± 2	13 ± 3	13 ± 3	17 ± 3	22 ± 2	27 ± 2
		[1.3]	[2.3]	[1.1]	[1.3]	[0.9]	[0.9]	[0.9]	[1.2]	[1.0]	[0.9]
	5,000	34 ± 2	63 ± 6	116 ± 12	137 ± 3	13 ± 1	13 ± 1	12 ± 2	20 ± 1	25 ± 2	23 ± 3
		[1.3]	[2.5]	[1.0]	[1.3]	[0.9]	[0.8]	[0.8]	[1.3]	[1.1]	[0.8]
Positive control^b^		225 ± 16	118 ± 8	465 ± 60	1504 ± 102	408 ± 12	142 ± 19	265 ± 20	182 ± 22	227 ± 25	104 ± 8
		[8.8]	[4.8]	[4.1]	[14.0]	[29.9]	[9.3]	[18.1]	[12.1]	[10.3]	[3.6]

Data are expressed as mean ± standard deviation. ^a^Three plates were used each dose. Factor = no. of colonies of treated plate/no. of colonies of negative control plate. ^b^TA98: 2-NF 2 *μ*g/plate (−S9 mix), B[a]P 1 *μ*g/plate (+S9 mix); TA100:SA 0.5 *μ*g/plate (−S9 mix), 2-AA 1 *μ*g/plate (+S9 mix); TA1535:SA 0.5 *μ*g/plate (−S9 mix), 2-AA 2 *μ*g/plate (+S9 mix); TA1537:ICR-191 0.5 *μ*g/plate (−S9 mix), 2-AA 1 *μ*g/plate (+S9 mix); and WP2 *uvr*A:4NQO 0.5 *μ*g/plate (−S9 mix), 2-AA 6 *μ*g/plate (+S9 mix). 2-NF, 2-nitrofluorene; B[a]P, benzo[a]pyrene; SA, sodium azide; 2-AA, 2-aminoanthracene; ICR-191, acridine mutagen ICR 191; and 4NQO, 4-nitroquinoline N-oxide.

**Table 7:** *In vitro* chromosome aberration test in Chinese hamster lung cells with EAG.

Treatment schedule^a^	S9 mix	Dose (*µ*g/mL)	PP + ER (%)	Ratio of aberrant metaphase^b^ (%)	Cell counts^c^		Mean	RICC^d^ (%)
					Flask A	Flask B		
06–18	+	0	0.00	0.00	8,662	8,264	8,463	100
		350	0.33	0.33	8,263	8,387	8,325	97
		700	0.67	0.67	8,563	8,790	8,676	104
		1,300	0.00	0.33	6,083	6,262	6,172	57
		1,400	0.00	0.00	5,440	5,382	5,411	43
		B[a]P 20	0.00	15.00^∗∗^	6,000	5,850	5,925	52

06–18	−	0	0.00	0.00	9,162	9,348	9,255	100
		300	0.33	0.00	9,528	8,722	9,124	98
		600	0.67	0.00	8,670	8,912	8,791	92
		1,100	0.00	0.33	6,609	6,657	6,633	57
		1,200	0.67	0.00	6,032	5,864	5,947	46
		4NQO 0.4	0.00	10.33^∗∗^	7,309	7,192	7,250	67

24–0	−	0	0.33	0.33	8,900	8,910	8,905	100
		225	0.67	0.00	8,996	9,273	9,134	104
		450	0.33	0.00	9,589	9,273	9,431	109
		800	1.00	0.67	6,245	6,552	6,398	56
		900	0.00	0.33	6,031	5,889	5,960	49
		4NQO 0.4	0.00	9.33^∗∗^	7,012	6,669	6,840	64

Initial cell count					3,142	3,162	3,152	

^∗∗^Significant difference at *P* < 0.01 levels compared with the negative control by Fisher's exact test. ^a^Treatment time – recovery time, hours, ^b^Gap excludes, 150 metaphases were examined per culture. ^c^After harvesting mitotic cells, each culture was trypsinized and suspended with 0.5 mL of 0.1% trypsin and 5 mL of culture medium. The cell suspensions of 0.4 mL/culture were diluted 50 times with 19.6 mL of Isoton® sol. The cells in 0.5 mL of Isoton® sol. were counted twice/culture using Coulter Counter model Z2. The actual number of cells per flask = mean cell count × 550. ^d^Relative increase in cell count = ((cell count of treated flask – initial cell count)/(cell count of the negative control flask – initial cell count)) × 100, PP, polyploid; ER, endoreduplication; B[a]P, benzo[a]pyrene (positive control); and 4NQO, 4-nitroquinoline-1-oxide (positive control).

**Table 8:** Observations of micronucleus and PCE:RBC ratio.

Test article	Dose (mg/kg/day)	Animals per dose	MNPCE^a^ (mean ± SD)	PCE:RBC ratio (mean ± SD)	% control
EAG	0	6	1.33 ± 1.03	0.57 ± 0.01	100
	500	5^b^	1.20 ± 0.84	0.58 ± 0.02	101
	1,000	6	1.00 ± 1.10	0.57 ± 0.02	100
	2,000	6	1.50 ± 1.38	0.57 ± 0.01	99

CPA	70	6	110.50 ± 29.71^∗∗^	0.39 ± 0.02^##^	69

^∗∗^Significant difference at *P* < 0.01 levels compared with the negative control by the Mann–Whitney. ^##^Significant difference at *P* < 0.01 levels compared with the control by Student's *t*-test. ^a^Ratio of MNPCE with 4,000 PCE, ^b^One of the mice was died. PCE, polychromatic erythrocyte; RBC, red blood cells (polychromatic erythrocyte + normochromatic erythrocyte); MNPCE, micronucleated polychromatic erythrocyte; and CPA, cyclophosphamide monohydrate (positive control).

## Data Availability

The data used to support the findings of this study are available from the corresponding author upon request.
